# The rate of use of Veterans Affairs chiropractic care: a 5-year analysis

**DOI:** 10.1186/s12998-022-00413-9

**Published:** 2022-01-21

**Authors:** Ryan Burdick, Kelsey L. Corcoran, Xiwen Zhao, Anthony Lisi

**Affiliations:** 1 Center for Medical Informatics, Yale School of Medicine, Yale University, 300 George St, New Haven, CT 06511 USA; 2 Department of Physical Medicine and Rehabilitation, VA Connecticut Healthcare System, 950 Campbell Ave., West Haven, CT 06516 USA; 3 Pain Research, Informatics, Multimorbidities, and Education (PRIME) Center, VA Connecticut Healthcare System, 950 Campbell Ave., West Haven, CT 06516 USA; 4 Center for Analytical Sciences, Yale School of Public Health, Yale University, 300 George St, New Haven, CT 06511 USA

**Keywords:** Veterans, Chiropractic, Non-pharmacologic, Community care, Pain management

## Abstract

**Background:**

The US Department of Veterans Affairs (VA) has initiated various approaches to provide chiropractic care to Veterans. Prior work has shown substantial increase in use of VA chiropractic care between fiscal years (FY) 2005–2016. However, the extent of the availability of these services to the Veteran population remains unclear. The purpose of this study was to analyze the rate of Veteran use of VA chiropractic services, both from on-site care at VA facilities and VA purchased care from community care providers. This study analyzed facility characteristics associated with chiropractic use by both care delivery mechanisms (on-site and in the community).

**Methods:**

Cross-sectional analyses of administrative data were conducted for FY 2014–2019. Data were obtained from VA’s Corporate Data Warehouse. The variables extracted included number of unique Veterans receiving VA chiropractic care on-site and in the community, total Veteran population of the VA facilities, size of the VA chiropractic workforce (measured as Full-Time Equivalent, FTE), and facility characteristics (geographic region and the facility complexity). Descriptive statistics, mixed model, and multivariant models were used to analyze data.

**Results:**

Use of VA chiropractic care increased over the six-year period for both on-site and community care. National average for on-site use of the population was 1.27% in FY14 and 1.48% in FY19. Community care use was 0.29% and 1.76% for the same years. Use at individual facilities varied widely in each FY. Factors such as chiropractor FTE, geographic locations, and the complexity of the VA facility are associated with use of chiropractic services.

**Conclusion:**

The VA has expanded the non-pharmacologic treatments available to Veterans by providing chiropractic services, yet chiropractic use remains low compared to other US populations. As Veterans have a high prevalence of pain and musculoskeletal conditions, continued work to assess and achieve the optimal levels of chiropractic use in this population is warranted.

## Introduction

The United States Department of Veterans Affairs (VA) provides healthcare services to over six million Veterans annually [1]. Most of this care, categorized as “on-station,” is delivered within VA’s national network of 170 medical centers and 1074 outpatient care facilities. Yet a growing amount is purchased from private sector facilities and/or providers through VA’s “community care” network [2]. Private sector care is purchased by the VA for many reasons, including when given services are not available in a timely manner at a VA facility or if a Veteran lives within a geographic area that does not provide on-station chiropractic care.

VA began to include Doctors of Chiropractic (DCs) among its community care network in 2000 and on-station at VA facilities in 2004, with increasing numbers of Veterans utilizing chiropractic care annually from both delivery mechanisms [2, 3]. DCs most commonly manage musculoskeletal (MSK) disorders such as low back or neck pain [4]. MSK conditions are highly prevalent within the Veteran population and are associated with increased opioid prescriptions [5]. The high prevalence of these pain conditions and the potential negative effects of prescription opioid use underscore the need to adhere to clinical practice guidelines—which recommend non-pharmacological therapies as frontline treatments—in the management of MSK conditions [6, 7]. Prior work has shown a negative association between chiropractic use and opioid prescriptions among Veterans of recent wars [8] and other United States populations [9].

In 2016, VA Health Services Research and Development State-of-the-Art Conference on Non-Pharmacological Treatments for Chronic Pain recommended the increased uptake of evidence-based non-pharmacological approaches by VA facilities [10]. Within these recommendations was chiropractic care, which includes many of the guideline recommended therapies, including but not limited to spinal manipulation, rehabilitative exercises, and pain education [11]. Also in 2016, the US Congress passed the Comprehensive Addiction and Recovery Act in light of the nation’s opioid epidemic [12]. This law led to a VA directive to address pain management for Veterans and to conduct research on the implementation and impact of complementary and integrative health (CIH) [13]. Furthermore, VA’s Office of Patient-Centered Care and Cultural Transformation formalized an approach to care called the Whole Health System of Care. This approach incorporates patient-centered care and CIH, including the use of chiropractic care at many VA facilities [14]. In March 2018, the United States’ Congress mandated further expansion of on-station chiropractic care within the VA, which was codified in May 2018 via VA Directive 1210 [15].

These initiatives have provided policy support for more Veterans to use VA chiropractic care. Prior work has shown a substantial increase in the number of Veterans using VA chiropractic care between fiscal years (FY) 2005–2016 [3]. However the proportion of the Veteran population using these services, and factors associated with their use, remains unclear. The purpose of this study was to measure the rate of use of VA chiropractic care in the Veteran population both via on-station care at VA facilities and community care mechanisms over a 5-year period, and to explore facility characteristics associated with chiropractic care use.

## Methods

The project was a program analysis of VA chiropractic care use for the 5 fiscal years (October 1 of a given calendar year through September 30 of the next) of 2014 to 2019, encompassing October 1, 2013 to September 30, 2019. As a program assessment work, the VA Connecticut Research and Development Office determined that this did not arise from research requiring Institutional Board Review.

Chiropractic care use was defined as the percent of patients using VA chiropractic care, via on-station or community care mechanisms as indicated, out of the total population of VA patients in a FY. All data were obtained from VA’s Corporate Data Warehouse, a central repository of administrative and clinical data, using previously validated methodology [3]. The count of on-station chiropractic patients was determined by the number of patients at each facility with a distinct social security number (SSN) with a visit associated with the chiropractic care identifier code (Stop Code) 436 in the primary or secondary position by FY. The number of facilities with on-station chiropractic care was determined by the count of facilities with any number of chiropractic patients in a given FY.

The number of VA chiropractic patients receiving community care was obtained from the VA Office of Community Care claims database. Chiropractic patients were identified by distinct SSN and by facility location.

We extracted facility information including VA district, facility medical complexity grouping (MCG), DC full time equivalent (FTE) staffing, and the total Veteran patients of each VA facility. [16] The VA district of each facility is based on VA’s 5 geographic districts. Facilities’ MCG ratings, a product of the VA Office of Productivity, Efficiency, and Staffing (OPES), are ordered from 1a, 1b, 1c, 2, and 3, with MCG 1a facilities representing the most complex facilities. These are based on criteria such as patient volume, case complexity, staffing levels, and dedication to education or research [17]. These classifications were extracted to investigate any potential geographic trends within the VA districts or if trends exist within the complexity of similarly categorized facilities. Chiropractor staffing was obtained as the sum of VA chiropractor employee clinical FTE and fee-for-service chiropractors working at VA facilities whose effort is converted to the FTE scale. Clinical FTE represents both the employment schedule of the chiropractor (i.e. full-time vs part-time) and the percentage of their work hours administratively assigned to clinical tasks, with a 1 representing a full-time employee whose work hours are 100% assigned to clinical care and less than a 1 representing an employee who works less than full-time and/or is labor mapped to additional activities besides patient care (i.e. research, administrative duties). DC FTE per total patients is the quotient of DC clinical FTE at each facility divided by the total Veteran patient population at that facility.

### Statistical method

Characteristics were summarized using frequencies (%) for categorical variables and means with standard deviations (SD) for continuous variables. The associations between facility characteristics and on-station and community care chiropractic use were analyzed via mixed effects models. A multivariable regression model assessed the use of chiropractic care adjusting for on-stations care use, community care use, FY, DC FTE per total patients, MCG level, and district. For the regression model of on-station chiropractic care use, only data from facilities with on-station chiropractic care were included. All statistical analyses were conducted using SAS software, Version 9.4 (SAS Institute Inc., Cary, NC, USA). Significance level was set at *p* < 0.05, two-sided.

## Results

**Fig. 1:**
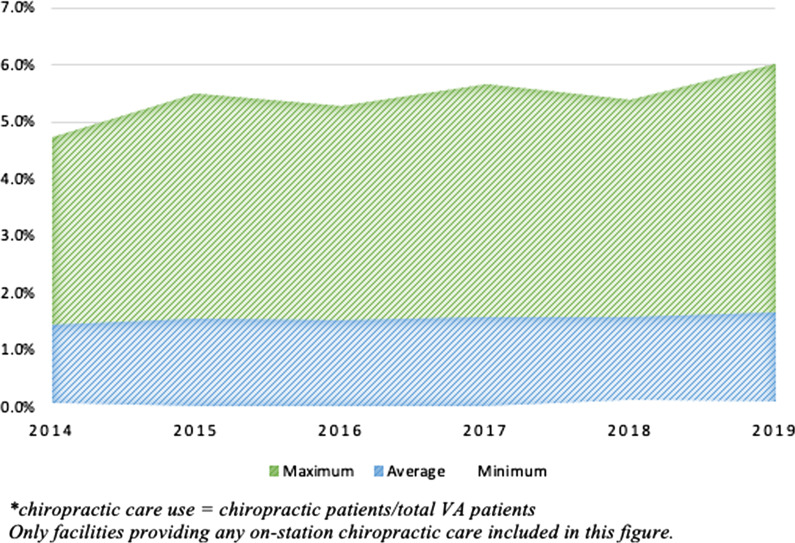
Range of on-station chiropractic care use* at VA facilities. *chiropractic care use = chiropractic patients/total VA patients. Only facilities providing any on-station chiropractic care included in this figure.

We identified 140 facilities that provided on-station chiropractic care during the study period. The chiropractic care use via on-station and community care, as well as national characteristics of VA chiropractic clinics, are presented in Table 1. The number of Veterans utilizing any VA health services increased by an average of 1% annually over the 6-year study period. Veterans receiving chiropractic care via on-station and community care increased by an average 18% and 53% per year respectively. There was wide variability in the use of on-station chiropractic care for each facility, which is presented in Fig. 1.

**Table 1:** VA population use of chiropractic services

FY	Total VA patients	On-station chiropractic care**				Community chiropractic care		
		VA chiropractic clinics	Mean clinic FTE	Chiropractic patients	Chiropractic care use*	Chiropractic patients	Chiropractic care use* (when VA clinic available)	Chiropractic care use* (when no VA clinic available)
2014	5,974,343	53	1.17	30,055	1.27%	18,125	0.32%	0.25%
2015	6,062,967	65	1.31	37,374	1.33%	28,332	0.53%	0.34%
2016	6,144,324	76	1.39	44,106	1.32%	25,072	0.68%	0.07%
2017	6,180,920	86	1.44	47,558	1.37%	46,216	0.54%	0.78%
2018	6,234,048	105	1.39	51,814	1.34%	83,548	1.01%	1.25%
2019	6,308,167	138	1.67	67,931	1.48%	130,928	1.49%	1.70%

FY = Fiscal Year; VA = Veterans Affairs; FTE = Full-time equivalent

^*^chiropractic care use = chiropractic patients/total VA patients

^**^Facilities providing any on-station chiropractic care included in calculation. Facilities without on-station chiropractic care were excluded

On-station chiropractic care use significantly increased over time at a rate of 4% per year (*p* = 0.002) after adjusting for community care use, DC FTE per total patients, MCG, and district (Table 2). Each 1% increase in community care use was associated with a 4% decrease of on-station chiropractic use (*p* = 0.02), and increasing DC FTE per total patients was associated with increasing on-station chiropractic use (*p* =  < 0.001).

**Table 2:** Factors associated with on-station chiropractic care use

Factor	Coefficient estimate	Standard error	*p*-value
Fiscal year	0.037	0.012	0.002
Community care use	− 0.043	0.019	0.022
DC FTE per total patients	0.696	0.031	< .0001
*MCG*			0.434
MCG 1a	*Ref*		
MCG 1b	0.030	0.161	0.852
MCG 1c	− 0.187	0.149	0.211
MCG 2	0.039	0.169	0.813
MCG 3	− 0.181	0.163	0.267
*District*			0.477
District 1	*Ref*		
District 2	0.097	0.185	0.600
District 3	0.039	0.136	0.776
District 4	-0.180	0.164	0.273
District 5	-0.144	0.170	0.397

DC FTE = Doctor of Chiropractic Full-time Equivalent; MCG = Medical Complexity Grouping

Community care use also increased significantly over time at a rate of 18% per year (*p* < 0.001) after adjusting for on-station use, DC FTE per total patients, MCG, and district (Table 3). Increasing DC FTE per total patients was associated with increasing care use (*p* = 0.04), while increasing on-station use by 1% was associated with a 25% decrease in community care use (*p* = 0.008). District was also associated with community care use (*p* = 0.001).

**Table 3:** Factors associated with community care chiropractic use

Factor	Coefficient estimate	Standard error	*P*-value
Fiscal year	0.183	0.017	< .0001
On-station use	− 0.250	0.094	0.008
DC FTE per total patients	0.188	0.091	0.038
*MCG*			0.050
MCG 1a	*Ref*		
MCG 1b	− 0.150	0.155	0.335
MCG 1c	− 0.019	0.141	0.891
MCG 2	0.331	0.159	0.037
MCG 3	0.175	0.147	0.234
*District*			0.010
District 1	*Ref*		
District 2	0.069	0.162	0.672
District 3	0.325	0.134	0.016
District 4	0.174	0.150	0.246
District 5	0.498	0.153	0.001

DC FTE = Doctor of Chiropractic Full-time Equivalent; MCG = Medical Complexity Grouping

## Discussion

This work presents the first description of VA chiropractic care as a percentage of the overall VA patient population. From FY 2014 to 2019, the percentage of Veterans using VA chiropractic care increased annually, reaching nearly 1.5% for on-station and 1.7% for community care in FY 2019. While previous work has shown the absolute number of VA patients receiving chiropractic care has increased annually [3], results of the current study show that the penetration of chiropractic care use in VA has also increased. Nationally, the mechanism (on-station or community care) where Veterans use chiropractic care is inversely associated. As VA continues to analyze the most effective and efficient mechanisms to deliver chiropractic care, further investigation into both care pathways is warranted.

Although the use of chiropractic care in VA has increased, it remains lower than that reported in other populations. Prior studies found that chiropractic care was used by approximately 13% of patients at Military Treatment Facilities [18] and approximately 15% of the general US population [19]. Since the Veteran population is known to have a high prevalence of MSK conditions [20], and treatments administered by DCs are aligned with primary approaches for the treatment of MSK conditions, the current use of VA chiropractic care may be suboptimal.

### On-station chiropractic use

Variations in on-station chiropractic care use were associated with facility characteristics. Lower rates of use were associated with higher complexity facilities based on the MCG model. The reason for this may be that chiropractic care is more readily used at facilities that have fewer overall options for managing MSK cases. The analysis found an association between allotted work force, measured as DC FTE per total patients, and on-station use. This suggests that in order for VA to provide increased availability of chiropractic care, an increase in the VA chiropractic workforce is necessary. Future research is needed to assess the best level of DC FTE and chiropractic care use as VA continues to expand services for Veterans.

### Community care chiropractic use

The VA district representing the western United States had the highest community care use, which is likely related to the highly rural areas in this region. Veterans in rural areas may have increase community care referrals based on their limitations in accessing a VA facility with an on-station chiropractic clinic. Nationwide, variations in community care may also be due to multiple third-party contractors coordinating VA’s community care services.

### Limitations

This project had several limitations. All administrative databases are subject to data transmission, storage, and retrieval error. However prior work has indicated that VA administrative data regarding on-station care are highly accurate in multiple patient parameters [21, 22]. If minor errors occurred, it seems unlikely that the data errors would substantially change the results with respect to on-station chiropractic care use. Data from VA’s community care network may be subject to regional variation in claims being associated with a purpose of visit indicated as chiropractic. The purpose of visit data may be inaccurate or missing, thus, our results in this category may be underreported. Within the community care data, there is a known limitation in FY15-16 where many claims were administratively process and attributed to one VA facility, resulting in incorrect distribution of services among VA facilities nationally. These data sources do not include any Veteran who utilized chiropractic care outside of the VA system and elected to self-pay, which could lead to the underreporting of chiropractic care use.

Additional work is needed to assess the optimal use of chiropractic care for Veterans. Future studies should explore details of chiropractic care patterns (timing, dosage, and sequencing) and the impacts of such care on patient level and systems outcomes. Additionally, studies comparing VA chiropractic care from on-station and community care providers could inform the ongoing implementation of services in a manner that maximizes outcomes for Veterans and resource allocation within the VA.

## Conclusions

The VA has expanded Veterans’ use of non-pharmacological treatments provided via chiropractic care within both on-station and community care settings, yet chiropractic use remains low compared to other US populations. As Veterans have high prevalence of pain and MSK conditions, continued work to assess and achieve the optimal levels of VA chiropractic care use in this population is warranted.

## Data Availability

To maximize protection security of veterans’ data while making these data available to researchers, the US Department of Veterans Affairs (VA) developed the VA Informatics and Computing Infrastructure (VINCI). VA researchers must log onto VINCI via a secure gateway or virtual private network connection (VPN), and use a virtual workspace on VINCI to access and analyze VA data. By VA Office of Research and Development policy, VINCI does not allow the transfer of any individual-level data out of its secure environment without special permission. Researchers who are not VA employees must be vetted and receive “without compensation” (WOC) employee status to gain access to VINCI. For questions about data access, contact the study lead (Ryan.Burdick@va.gov) or the VA Office of Research and Development (VHACOORDRegulatory@va.gov).

## Abbreviations

VA: United States Department of Veterans Affairs
DC: Doctor of chiropractic
MSK: Musculoskeletal
CIH: Complementary and integrative health
FY: Fiscal year
VSSC: VA Support Service Center
FTE: Full time equivalent
MCG: Medical complexity grouping
OPES: VA Office of Productivity, Efficiency, and Staffing

## References

[1] Affairs UDoV. VA Benefits and Health Utilization 2021. https://www.va.gov/vetdata/docs/pocketcards/fy2021q4.pdf.

[2] OCC U. Veteran Community Care Eligibility Fact Sheet. In: US Department of Veteran Affairs OoCC, editor. 2019.

[3] Lisi AJBC (2016). Trends in the use and characteristics of chiropractic services in the Department of Veterans Affairs. J Manipulative Physiol Ther.

[4] Beliveau PJH WJ, Sutton DA, Simon NB, Bussières AE, Mior SA, French SD. The chiropractic profession: a scoping review of utilization rates, reasons for seeking care, patient profiles, and care provided. Chiropractic Manual Therapy. 2017;25(35). 10.1186/s12998-017-0165-8 PMC5698931 29201346

[5] Edlund MJ (2014). Patterns of opioid use for chronic noncancer pain in the Veterans Health Administration from 2009 to 2011. Pain.

[6] Carpenter RWLS, Bruehl S, Trull TJ (2019). Concurrent and lagged associations of prescription opioid use with pain and negative affect in the daily lives of chronic pain patients. J Consult Clin Psychol.

[7] Qaseem AWT, McLean RM, Forciea MA (2017). Nonivasive treatments for acute, subacute, and chronic low back pain: a clinical practice guideline from the American College of Physicians. Ann Intern Med.

[8] Lisi ACK (2018). Opioid use among veterans of recent wars receiving veterans affairs chiropractic care. Pain Med.

[9] Corcoran KLBL, Gunderson CG, Steffens C, Brackett A, Lisi AJ (2020). Association between chiropractic use and opioid receipt among patients with spinal pain: a systematic review and meta-analysis. Pain Med.

[10] Kligler B ea. Clinical policy recommendations from the VHA state-of-the-art conference on non-pharmacological approaches to chronic musculoskeletal pain. J Gen Intern Med. 2018;33:16–23. 10.1007/s11606-018-4323-z PMC5902342 29633133

[11] Hartvigsen JFS. So, what is chiropractic? Summary and reflections on a series of papers in Chiropractic and Manual Therapies. Chiropr Man Therap. 2020;28(1). 10.1186/s12998-019-0295-2 PMC6990530 32000811

[12] Rudd RA, Aleshire N, Zibbell JE, Gladden RM (2016). Increases in drug and opioid overdose deaths-United States, 2000–2014. MMWR Morb Mortal Wkly Rep.

[13] VHA Directive 1137. In: Affairs UDoV, editor. 2017.

[14] Bokhour BG HJ, Zeliadt S, Mohr DC Whole Health System of Care Evaluation- A Progress Report on Outcomes of the WHS Pilot at 18 Flagship Sites. In: Veterans Health Administration CfEP-CCiV, editor. https://www.va.gov/WHOLEHEALTH/professional-resources/clinician-tools/Evidence-Based-Research.asp2020.

[15] VHA Directive 1210 Chiropractic Care. In: Affairs UDoV, editor. 2018.

[16] map VD. https://www.va.gov/opal/sac/mspvng.asp.

[17] National Academies of Sciences E, and Medicine; Division of Behavioral and Social Sciences and Education; Board on Human-Systems Integration; Division on Engineering and Physical Sciences; Board on Infrastructure and the Constructed Environment; Committee on Facilities Staffing Requirements for Veterans Health Administration. Facilities Staffing Requirements for the Veterans Health Administration-Resource Planning and Methodology for the Future. Nature of Veterans Health Administration Facilities Management (Engineering) Tasks and Staffing. Washington DC: National Academies Press; 2019. 32293829

[18] Williams VF CL, McNellis MG. Use of complementary health approaches at military treatment facilities, active component, U.S. Armed Forces, 2010–2015. MSMR. 2016;23 (7):9–22. 27501938

[19] Weeks WBGC, Meeker WC, Marchiori DM (2015). Public perceptions of doctors of chiropractic: results of a national survey and examination of variation according to respondents' likelihood to use chiropractic, experience with chiropractic, and chiropractic supply in local health care markets. J Manipulative Physiol Ther.

[20] Nahin R (2016). Severe pain in veterans: the effect of age and sex, and comparisons with the general population. J Pain.

[21] Noel PHCL, Perrin RA (2010). VHA Corporate Data Warehouse height and weight data: opportunities and challenges for health services research. J Rehabil Res Dev.

[22] Miller DRPL (2008). Longitudinal approaches to evaluate health care quality and outcomes: the Veterans Health Administration diabetes epidemiology cohorts. J Diabetes Sci Technol.

